# Emergency embolization of a massive life-threatening placenta percreta bleeding following delivery by Cesarean section

**DOI:** 10.1016/j.radcr.2022.08.043

**Published:** 2022-09-28

**Authors:** Ilia A. Panfilov, Alexander Venmans, Hans G. Kortman, Peter F. Boekkooi, Cora A. Fiedeldeij, Paul N.M. Lohle

**Affiliations:** a Department of Radiology*,* Section Interventional Radiology*,* Elisabeth Tweesteden Ziekenhuis, Hilvarenbeekse Weg 60, Tilburg, 5022 GC, The Netherlands; b Department of Gynaecology, Elisabeth Tweesteden Ziekenhuis, Tilburg, The Netherlands

**Keywords:** Placenta accreta, Placenta percreta, Embolization, Bleeding, Blood loss, Uterus, CS, Cesarean sections, FIGO, Fédération Internationale de Gynécologie et d'Obstétrique, US, ultrasound, UAE, uterine artery embolization, HCG, Human chorionic gonadotropin

## Abstract

The rise in the number of Cesarean sections (CS) worldwide has increased the incidence of the placenta accreta spectrum disorders in the past years. About 5% of patients undergoing a CS develop placenta percreta. A 30-year-old woman, G2P1 with previous uncomplicated CS delivery had an elective CS delivery at 37w6d. The delivery was complicated by a substantial hemorrhage. On emergency laparotomy a placenta percreta was seen in the broad ligament, which could not be removed surgically. Embolization was performed with Gelfoam particles until stasis in the right uterine artery with placement of a coil. Patient discharge was 12 days after intervention. Emergency embolization is an effective treatment in bleeding complications due to placenta percreta at partus.

## Introduction

The rise in number of Cesarean sections (CS) worldwide has increased the incidence of the placenta accreta spectrum disorders in the past years. About 5% of patients undergoing a CS develop placenta percreta [1,2]. The underlying pathophysiologic mechanism entails the placenta growing beyond the endometrium into the surrounding tissues, causing severe and possibly fatal hemorrhage at delivery. This condition is classified by Fédération Internationale de Gynécologie et d'Obstétrique (FIGO) as the most severe of the placenta accreta spectrum disorders and typically develops in patients that have previously had uterine scarring by CS, curettage, or other uterine surgery [3].

Prenatal ultrasound (US) has increased the early detection and multidisciplinary treatment of this high-risk patient group. Most prevalent placenta percreta develop in the anterior uterine wall, related to the anterior incision of the uterus when CS is performed.

Anterior placenta percreta is usually diagnosed with obstetric US due to abnormalities of the bladder-myometrium interface with increased vascularity at the interface of the uterus and bladder.

Posterior placenta percreta involving of the broad ligament and parametrium is rarely reported in literature [4], [5], [6].

Failure to recognize this serious placental abnormality precludes us from making the appropriate plan for the delivery and consequently can lead to fatal results.

We describe a case of a patient with dorsolateral placenta percreta, which was not diagnosed during a planned CS and which was treated by emergency uterine artery embolization (UAE) for acute severe bleeding following subtotal hysterectomy.

## Case

A 30-year-old woman, gravida 2, para 1, with one previous uncomplicated CS delivery. Her medical history reported a gastric bypass. On prenatal ultrasound (US) at 20 weeks, there seemed to be an abnormal placentation on the right anterior and posterior/lateral uterus wall, and also in front of the cervix. The region of the uterine scar seemed unaffected. The ultrasound was repeated and in the 3^rd^ trimester the placenta was at 15 mm from the ostium interna with a posterior position. There were no signs of placenta accreta spectrum disorders. There was a hypoechogenic zone visible behind the placenta, and there were no lakes present. It was considered a placenta previa in a scarred uterus with the fetus in breech position. The case was discussed at a multidisciplinary consultation and a CS delivery was planned at 37w6d under spinal anesthesia. The risks of possible bleeding, UAE or hysterectomy were discussed with the patient, and informed consent was obtained.

Two gynecologists took part in the operation (PFB; CAF). Blood was pre-ordered for mother and child. Interventional radiology team was stand-by. After Pfannenstiel incision, delivery of the fetus was smooth without incision of placental tissue. However, the mother started bleeding profusely straight after the successful delivery. Manual removal of the placenta was unsuccessful due to complete adhesion to the posterior lateral wall and a placenta percreta was diagnosed. The patient and her partner were informed that emergency hysterectomy was needed under general anesthesia.

Inspection and palpation of the uterus demonstrated no signs of placental ingrowth into the urinary bladderwall or gastrointestinal tract. During surgery, it became clear that the earlier noted placenta percreta was located in the posterior and lateral wall of the uterus extending in the broad ligament. Subtotal hysterectomy was carried out above the level of the cervix. After clamping the uterine ligaments on the right side, it became clear that a large placental portion extended through the broad ligament and was heavily fixed to the parametrium on the right side. Blood loss continued up to 3000 mL and the patient became unstable requiring massive transfusion and noradrenalin. Both iliac arteries were compressed, and the interventional radiologist (PNML) was called for assistance in the operation room for an emergency UAE, since it was not technically possible to remove the placental mass in the broad ligament.

**Fig. 1:**
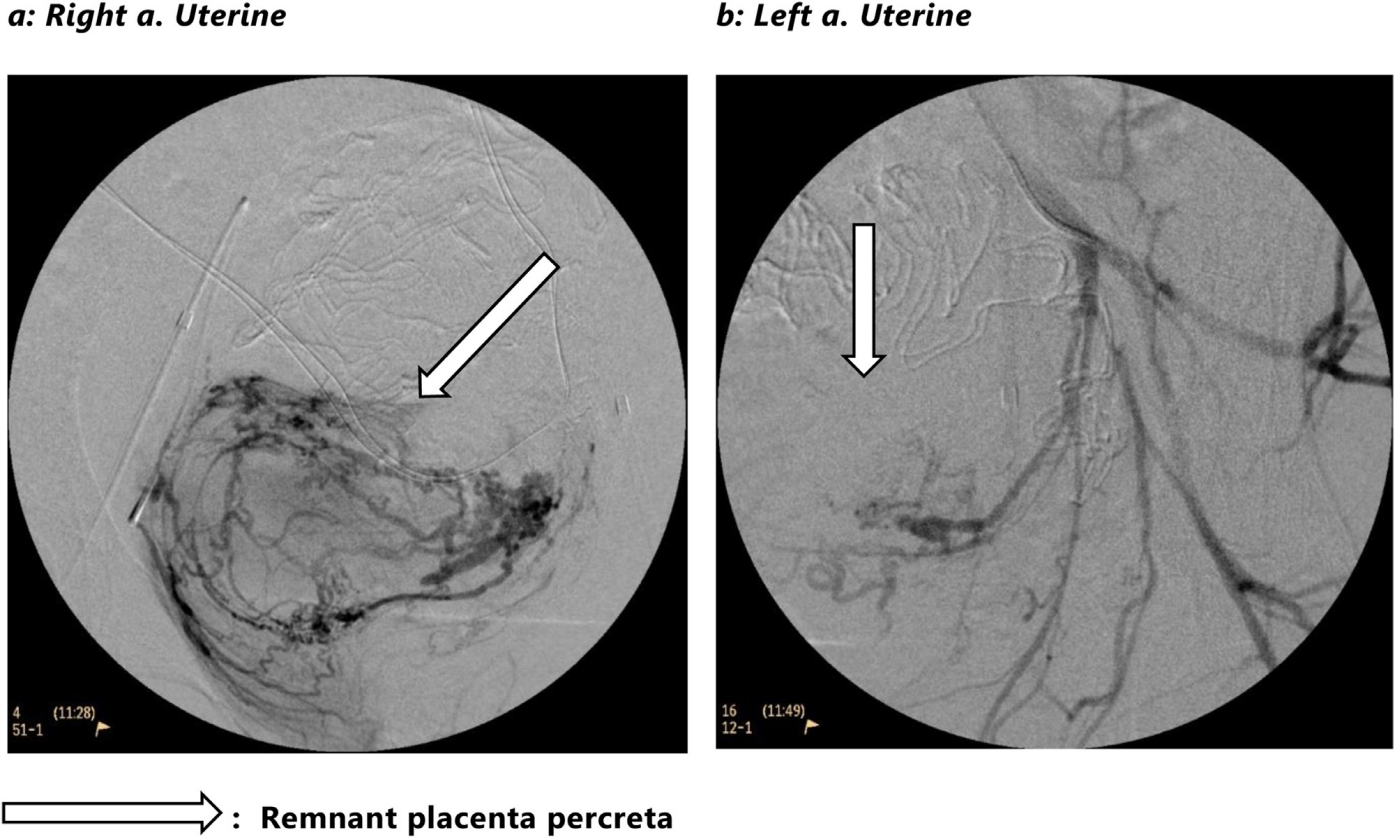
Embolization after hysterectomy.

Vascular access was obtained through the right femoral artery. Using selective angiography, the anterior division of the right internal iliac artery, the uterine artery remnant with distal arterial branches (corkscrew-like vessel branch appearance) were depicted directed toward a “uterine-like” mass (Fig. 1). Embolization was performed with Gelfoam particles (EmboCube 5.0 mm x 25mg, Merit©) until stasis in the right uterine artery with coil placement (VortX 18, Boston Scientific©) proximal in the right uterine artery. A similar procedure was performed on the left side. Control angiography demonstrated no contrast extravasation with complete stasis of contrast in both uterine artery remnants. Gynecological examination revealed no more evidence of bleeding upon inspection. Packing threads and gauzes were removed, and the abdomen was closed.

The total blood loss of 9000 mL required 12 units erythrocytes, 4 units thrombocytes and 12 units omniplasma. The patient was kept intubated and transported to the intensive care unit. Vital parameters stayed stable, and the patient was successfully detubated the day after. The newborn had normal development p40 and 2900 grams with a good start.

**Fig. 2:**
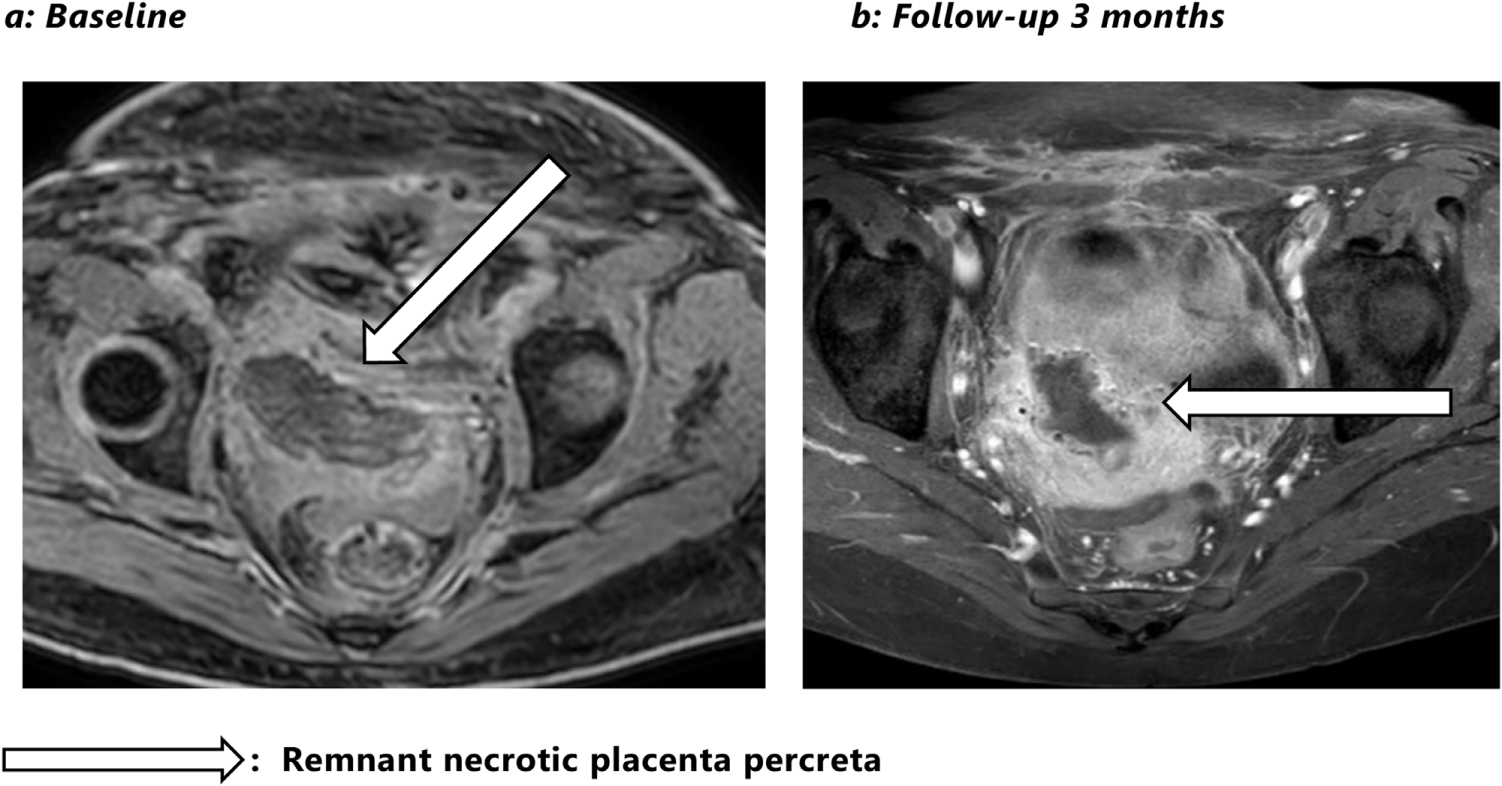
(A) Baseline and (B) follow-up at 3 months.

Contrast-enhanced MRI was performed 5 days after surgery to assess the extent of the placental remnant. A non-enhancing mass was seen in the obturator lodge of 125cc (Fig. 2A) with possible obstruction of the right ureter. There were no signs of placental percreta invasion into the bladderwall or gastrointestinal tract. A double-J catheter was placed in the right ureter. Human chorionic gonadotropin (HCG) levels dropped from 500 to 100. The patient and her newborn daughter were discharged from hospital 12 days after delivery with antibiotics. There were no readmissions and HCG dropped to 1 on day 24. Transvaginal inspection showed a normal cervix and transvaginal US showed further mass reduction (85%) down to 19cc. US at 3 months revealed even more mass reduction (92%) to 10cc (Fig. 2B).

## Discussion

This is the first reported case of an acute life-threatening massive placental percreta bleeding following CS successfully treated by emergent UAE after subtotal hysterectomy.

With regard to placenta accrete spectrum patients a meta-analysis reported high maternal mortality [1,7]. Several methods of treatment have been suggested when placenta percreta is diagnosed [8]. Treatment options are elective surgical removal of the uterus with the involving placental tissue, conservative management, or recently embolization prior to delivery. However, in case of massive acute life-threatening bleeding complication caused by the presence of an undiagnosed/unexpected placenta percreta, many gynecologists will resort to emergent hysterectomy as primary life-saving solution. Understandably, such massive bleeding complications requires quick decision-making and prompt surgical intervention, in which the area is difficult to oversee due to the presence of massive volumes of blood in the pelvis.

The dorso-laterally localized placenta percreta, as presented here, is much less common than localized elsewhere in the uterus. Because of overlapping structures and a deeper location in the pelvis, the dorso-laterally localized placenta percreta is difficult to diagnose with ultrasound and therefore often presented in an emergency setting [9]. Similar reported cases in literature were treated by conservative treatment and blood transfusion [4], [5], [6].

This is the first published case treated by selective UAE of the remaining placenta percreta with rapid recovery of the patient without complications.

Placenta percreta with extension into the broad ligament and parametrium follows the vasculature of the uterine artery and be suitable for UAE. The main advantage of UAE is deep penetration in the remaining placental tissue and thereby stops the bleeding.

## Conclusion

Massive life-threatening bleeding complications due to a dorso-lateral located placental percreta with broad-ligament and parametrial invasion can be treated successfully with emergency UAE.

## Take-home points

Emergency embolization is an effective treatment in bleeding complications due to placental percreta at partus.

## Author contributions

PFB – took part in the cesarean sections, patient is under care of this physician; CAF – took part in the cesarean sections, patient is under care of this physician; IAP – prepared the manuscript; PNML – took part in the intervention, reviewed and edited technical details of the performed procedure; AV – took part in the intervention, reviewed and edited technical details of the performed procedure; HGK - prepared the manuscript

All authors read and approved the final manuscript.

## Ethics approval and consent to participate

Ethics approval was waived in this retrospective case report.

## Patient consent

Written informed consent was obtained from the patient for publication of this case report and accompanying images.
